# Blue light inactivation of the enveloped RNA virus Phi6

**DOI:** 10.1186/s13104-021-05602-y

**Published:** 2021-05-17

**Authors:** Petra Vatter, Katharina Hoenes, Martin Hessling

**Affiliations:** Institute of Medical Engineering and Mechatronics, Ulm University of Applied Sciences, Ulm, Germany

**Keywords:** Phi6, Photoinactivation, Blue light, Coronavirus, SARS-CoV-2

## Abstract

**Objective:**

Ultraviolet radiation is known for its antimicrobial properties but unfortunately, it could also harm humans. Currently, disinfection techniques against SARS-CoV-2 are being sought that can be applied on air and surfaces and which do not pose a relevant thread to humans. In this study, the bacteriophage phi6, which like SARS-CoV-2 is an enveloped RNA virus, is irradiated with visible blue light at a wavelength of 455 nm.

**Results:**

For the first time worldwide, the antiviral properties of blue light around 455 nm can be demonstrated. With a dose of 7200 J/cm^2^, the concentration of this enveloped RNA virus can be successfully reduced by more than three orders of magnitude. The inactivation mechanism is still unknown, but the sensitivity ratio of phi6 towards blue and violet light hints towards an involvement of photosensitizers of the host cells. Own studies on coronaviruses cannot be executed, but the results support speculations about blue-susceptibility of coronaviruses, which might allow to employ blue light for infection prevention or even therapeutic applications.

## Introduction

Since December 2019, a new coronavirus capable of causing the severe pulmonary infection CoVid-19 has been spreading worldwide, and is therefore referred to as SARS-CoV-2 (severe acute respiratory syndrome coronavirus). As the number of infected and fatalities continues to rise, with more than 100 million infections and more than 2 million fatalities at the beginning of February 2021 [1], disinfection options are being sought to contain the further spread of the virus. Chemical disinfectants, heat, and ultraviolet radiation are successful against the virus [2–6], but can also be harmful to humans.

In recent years, visible blue and violet light has been employed to inactivate bacteria and fungi without particularly harming human cells [7–16]. The mechanism of action, which is similar for prokaryotic and eukaryotic cells, is based on endogenous photosensitizers naturally occurring in these microorganisms, such as porphyrins or flavins [17–23]. These photosensitizers absorb visible light of specific wavelengths and generate so-called reactive oxygen species (ROS) in the presence of oxygen, including ^1^O_2_, O_2_^*−^, H_2_O_2_ and HO^*^, which attack and kill the cells from inside.

Initial studies reveal that violet light with a wavelength of 405 nm has an inactivating effect on viruses [24–26]. This is even true for the bacteriophage phi6, which, like the SARS-CoV-2 virus, is an enveloped RNA virus [27]. Therefore, it is hoped that SARS-CoV-2 is also sensitive to violet light.

Studies on the effect of blue, non-violet light on any viruses do not exist so far, although this wavelength range (450–470 nm) has advantages over violet light. It is even less harmful to human cells [9–11, 28] and exhibits a higher penetration depth into human tissue, which might lead to future local therapies that try to fight coronaviruses in the human body, if coronaviruses exhibit a sensitivity to visible light. At least, some local blue or violet illumination applications have been investigated for the treatment of bacterial or fungal infections, e. g. as therapy for acne [29, 30], *Helicobacter pylori* infections in the stomach [31], vaginal infections [32] and for the prevention of ventilator-associated pneumonia [33].

Unfortunately, we are not allowed to work with coronaviruses in our laboratory. Therefore, in the study presented here, experiments on the inactivation of phi6—as a non-pathogenic coronavirus surrogate—are performed with 455 nm blue light and compared to the results of a previous 405 nm investigation [27].

## Main text

### Method

#### Irradiation setup

**Fig. 1:**
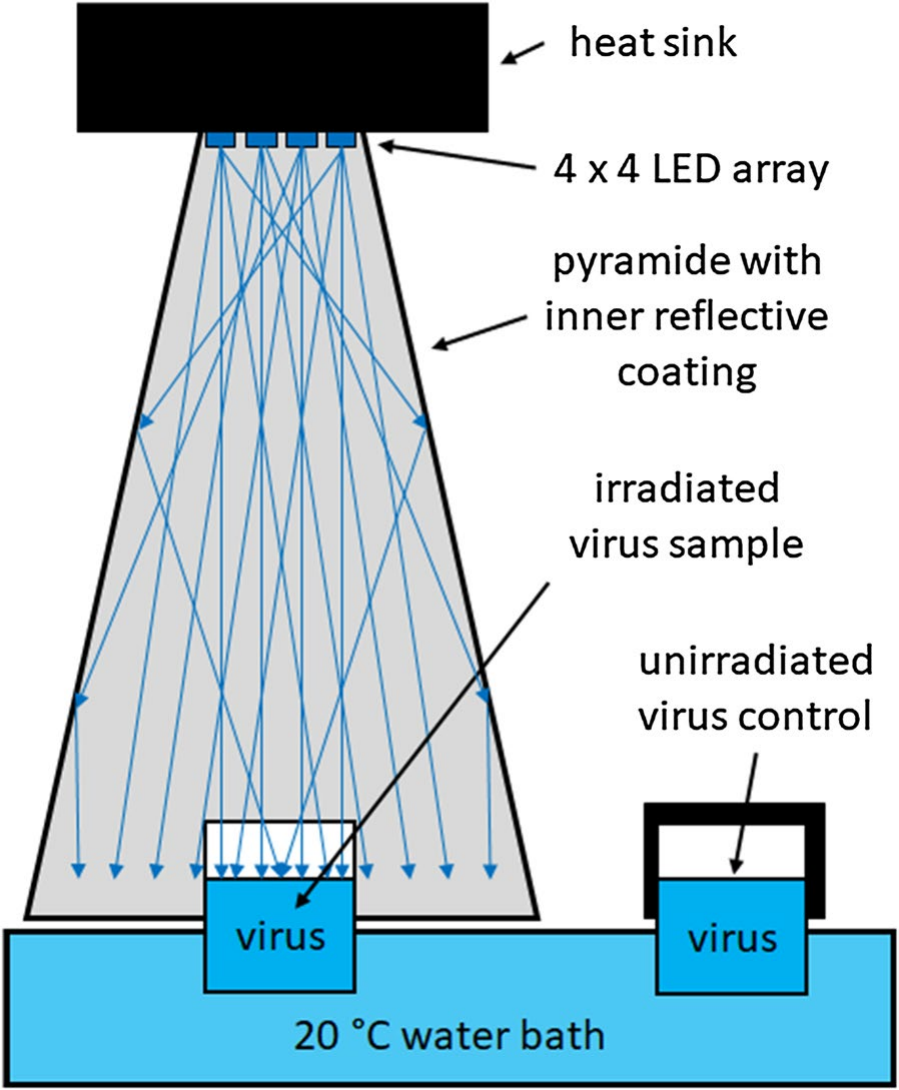
Scheme of the illumination setup

**Fig. 2:**
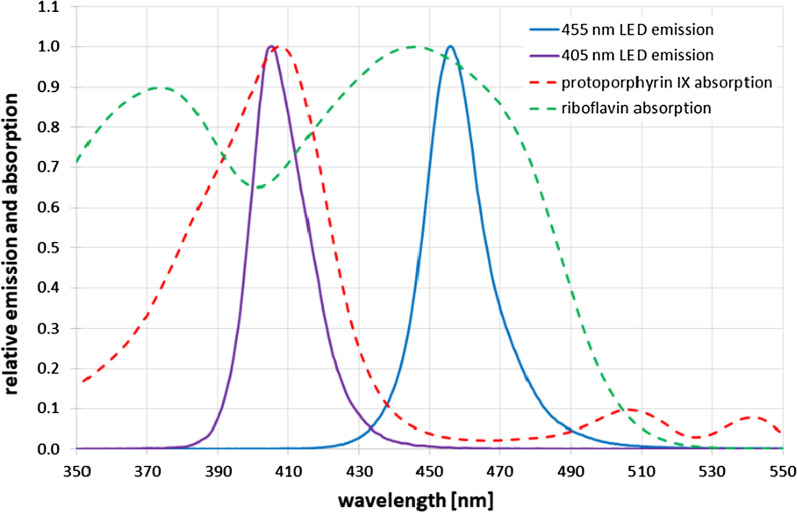
455 nm LED emission spectrum, with additional spectrum of the 405 nm LED of [27] and typical bacterial (!) photosensitizer absorption spectra [34] for comparison

The description of the irradiation setup is presented schematically in Fig. 1. Two glass beakers containing a virus-containing solution are kept at approximately 20 °C using a temperature-controlled water bath. One of the samples is irradiated from above by an array of 16 (4 × 4) 455 nm LEDs of RP-Technik GmbH (Rodgau, Germany). A hollow pyramid with reflective coating at the inside provides a homogeneous irradiance of up to 50 mW/cm^2^ in the sample plane at a distance of 28 cm. The emission spectrum of the employed 455 nm LEDs is given in Fig. 2, together with the emission spectrum of the 405 nm LED employed by Vatter et al. in a former study [27] and the absorption of known bacterial photosensitizers. This illustrates that at least for bacteria 405 nm and blue 455 nm irradiation involve different photosensitizers. The second beaker glass is shielded from light and serves as a control.

#### Microbiological experiments

Test virus is the bacteriophage phi6 (DSM 21518), which is multiplied by its host bacterium *Pseudomonas syringae* (DSM 21482). For the experiments, approximately 1.5 × 10^7^ viruses or plaque forming units (PFU) per ml of a phosphate buffered saline (PBS) solution are prepared. Samples are drawn for 0 h, 8 h, 16 h, 24 h, 32 h and 40 h of irradiation. At the end of each irradiation experiment, a double agar overlay plaque assay is performed: small volumes of the irradiated and non-irradiated virus samples are first mixed with *Pseudomanas syringae* and then poured as a liquid agar layer onto solid agar plates. In the absence of replicable viruses, bacteria will multiply in the agar and provide detectable turbidity. However, existing phi6 can infect and lyse bacteria. This creates holes/plaques in the agar turbidity from which the concentration of replicable phi6 in the samples and thus the disinfection effect of the 455 nm radiation can be calculated [27, 35].

### Results

**Fig. 3:**
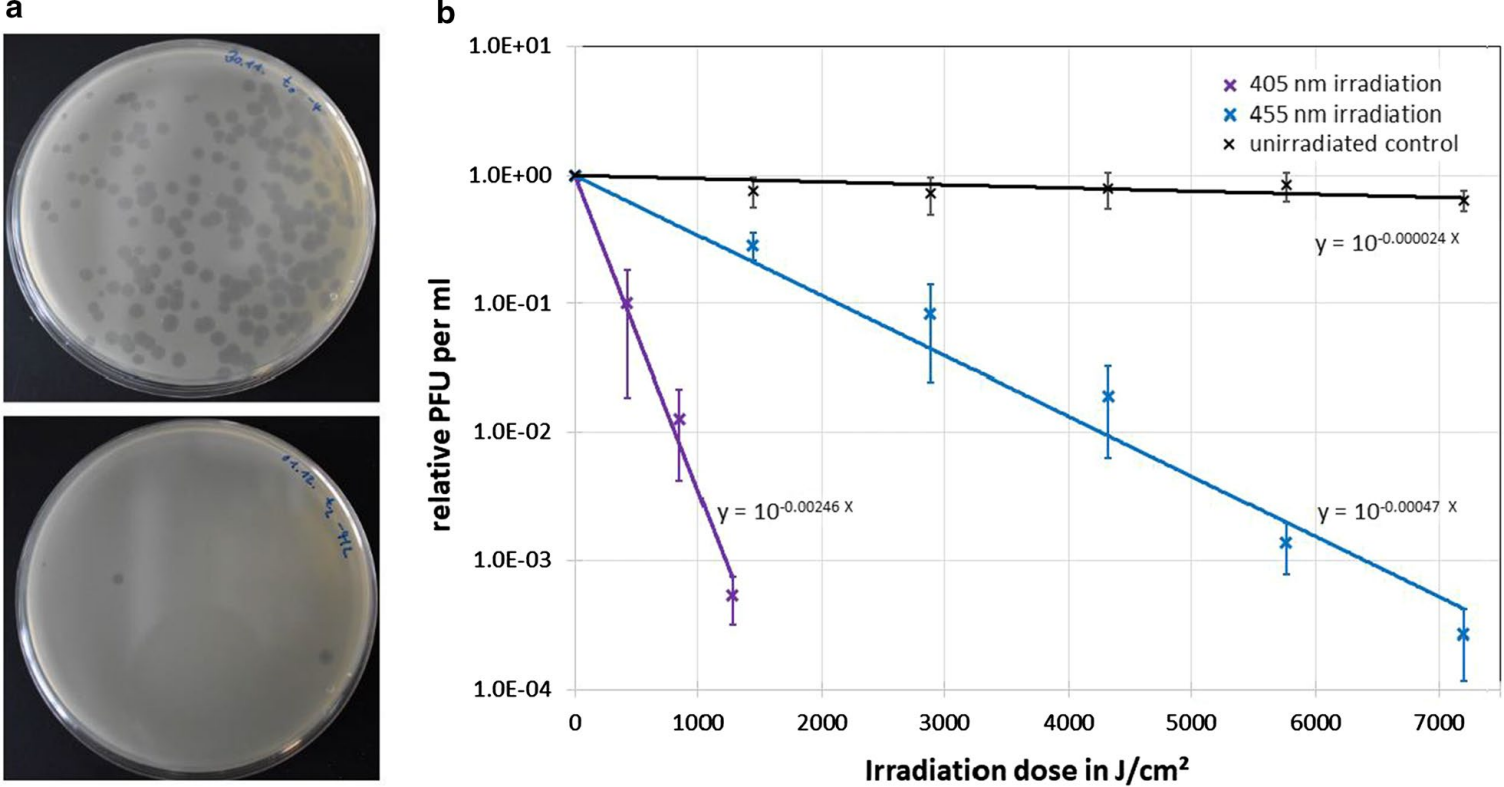
Results of the 455 nm irradiation of phi6 samples. **a** Example photographs of virus solution on agar plates. After 24 h the viruses have created visible plaques in the bacterial lawn. Top: non-irradiated sample, bottom: same sample after 2880 J/cm^2^ at 455 nm. **b** Evolution of phi6 concentration in plaque forming units (PFU) per ml as a function of the 455 nm irradiation dose. The former 405 nm results [27] are added for comparison. Each value represents the average of at least three independent experiments and the error bars depict the standard deviation of these single measurements

At least three technical replicates were performed of each individual irradiation dose up to 7200 J/cm^2^ over a period of up to 40 h and each series of measurements was executed three times. Typical results for an non-irradiated and an irradiated virus sample with the double agar overlay plaque assay are illustrated in Fig. 3. The difference in the number of plaques—and therefore viruses—between non-irradiated and irradiated sample is evident. Figure 3b reveals the quantitative results. The phi6 concentrations in the non-irradiated samples hardly changed during the 40 h duration of the experiment, but in the irradiated samples the virus concentration was successfully reduced by more than three orders of magnitude after 7200 J/cm^2^ at 455 nm. The necessary log-reduction dose is about 2130 J/cm^2^ at 455 nm—compared to a log-reduction dose of approximately 430 J/cm^2^ at 405 nm according to Vatter et al. [27].

### Discussion

For the first time, it could be demonstrated that the enveloped RNA virus phi6 is sensitive to visible blue light with a wavelength of 455 nm. The sensitivity is about 5 times lower than its 405 nm sensitivity, which was observed in a previous study [27]. This ratio is a typical sensitivity ratio known for pseudomonads and bacteria in general between these wavelengths [36, 37].

Nevertheless, the virus sensitivity to visible light is unexpected. In bacteria the presence of endogenous photosensitizers like porphyrins and flavins is well known, because they are results of the bacterial metabolism. The virus however exhibits no metabolism and should not need or produce such photosensitizers. Even if it contains one photosensitizer this should be effective either at 405 nm or 455 nm—but not at both wavelengths. The fact that the virus concentration is reduced at both wavelengths and the sensitivity ratio between 455 and 405 nm, which is similar to the above mentioned typical bacterial ratios, gives room for the speculation that the virus unintentionally takes along the bacterial photosensitizers of its host (*Pseudomomas syringae*) when it assembles its envelope.

## Conclusion

Whether the more important SARS-CoV-2 also contains photosensitizers and exhibits photoinactivation sensitivity towards blue or visible light is unknown so far, but there are hints that this coronavirus might at least contain porphyrins [38], which would possibly result in a sensitivity towards 405 nm irradiation. The advantage of a 455 nm light sensitivity could be the higher penetration depth of blue light in human tissue compared to violet light in a—speculative—future antiviral therapy.

## Limitations

Coronaviruses and phi6 are both enveloped RNA viruses, and phi6 has often been applied as coronavirus surrogate in the past, but so far there is no prove for any sensitivity of coronaviruses towards blue or violet light. Unfortunately, our lab does not have the required security clearance for coronavirus experiments.

## Data Availability

All data generated or analyzed during this study are included in this published article.

## Abbreviations

LED: Light emitting diode
SARS-CoV-2: Severe acute respiratory syndrome coronavirus 2
CoVid 19: Covid coronavirus disease 2019
RNA: Ribonucleic acid
ROS: Reactive oxygen species
LED: Light emitting diode
DSM: Deutsche Sammlung von Mikroorganismen und Zellkulturen
PBS: Phosphate buffered saline
